# Microarray analysis of ductal carcinoma in situ samples obtained by puncture from surgical resection specimens

**DOI:** 10.1186/s13104-021-05760-z

**Published:** 2021-08-30

**Authors:** Tomoo Jikuzono, Eriko Manabe, Shoko Kure, Haruki Akasu, Tomoko Ishikawa, Yoko Fujiwara, Masujiro Makita, Osamu Ishibashi

**Affiliations:** 1 Department of Endocrine Surgery, Nippon Medical School, 1-1-5 Sendagi, Bunkyo-ku, Tokyo, 113-8602 Japan; 2 Department of Breast Surgery, Nippon Medical School Musashi Kosugi Hospital, 1-396 Kosugi-cho, Nakahara-ku, Kawasaki, 211-8533 Japan; 3 Department of Integrated Diagnostic Pathology, Nippon Medical School Musashi Kosugi Hospital, 1-396 Kosugi-cho, Nakahara-ku, Kawasaki, 211-8533 Japan; 4 Department of Integrated Diagnostic Pathology, Nippon Medical School, 1-1-5 Sendagi, Bunkyo-ku, Tokyo, 113-8602 Japan; 5 Department of Endocrine Surgery, Nippon Medical School Musashi Kosugi Hospital, 1-396 Kosugi-cho, Nakahara-ku, Kawasaki, 211-8533 Japan; 6 Department of Human Nutrition, Seitoku University, 550 Iwase, Matsudo, Chiba 271-8555 Japan; 7 Institute for Human Life Innovation, Ochanomizu University, 2-1-1 Otsuka, Bunkyo-ku, Tokyo, 112-8610 Japan; 8 Laboratory of Biological Macromolecules, Department of Applied Life Sciences, Graduate School of Life & Environmental Sciences, Osaka Prefecture University, 1-1 Gakuen-cho, Sakai, Osaka 599-8531 Japan

**Keywords:** Ductal carcinoma in situ (DCIS), Microarray analysis

## Abstract

**Objective:**

The incidence of ductal carcinoma in situ (DCIS) is increasing due to more widespread mammographic screening. DCIS, the earliest form of breast cancer, is non-invasive at the time of detection. If DCIS tissues are left undetected or untreated, it can spread to the surrounding breast tissue. Thus, surgical resection is the standard treatment. Understanding the mechanism underlying the non-invasive property of DCIS could lead to more appropriate medical treatments, including nonsurgical options.

**Data description:**

We conducted a microarray-based genome-wide transcriptome analysis using DCIS specimens obtained by puncture from surgical specimens immediately after surgery.

## Objective

Mammographic screening has led to an increase in early-stage breast cancer detection [1]. Ductal carcinoma in situ (DCIS) is characterised by the presence of abnormal cells in the milk duct of the breast and is considered the earliest form of breast cancer [2]. DCIS does not spread from its site of origin; therefore, it is non-invasive at the time of detection.

DCIS is a highly treatable Stage 0 breast cancer with a good prognosis. However, if DCIS tissues are left untreated or undetected, they can spread into the surrounding breast tissue. The standard treatment is surgical resection, which prevents the local recurrence and future invasion of DCIS. However, surgical resection of DCIS does not reduce the risk of death from breast cancer [3]. Although some clinical studies have evaluated non-surgical treatment of DCIS, they fail to provide evidence against resection as the standard of care [4]. Resection remains the standard treatment for two key reasons. First, DCIS is non-invasive at the time of detection, but it may progress to invasive carcinoma over time [5, 6]. Second, since a core-needle biopsy involves the collection of tissues from the site of a lesion only, cancer cells can be missed, resulting in a DCIS misdiagnosis [7].

In this study, surplus specimens of breast fine needle aspiration cytology were analysed. The DCIS specimens were obtained from patients who underwent surgery at Nippon Medical School Musashi Kosugi Hospital. Conventional ultrasound-guided biopsy was not performed in this study, but specimens were obtained by puncture from the resected tissues immediately after surgery. We performed genome-wide transcriptomic profiling using the Affymetrix Clariom D Assay (Thermo Fisher Scientific, Waltham, MA, USA), a next-generation microarray with more than 6 million probes, including unidentified transcripts. Six DCIS samples (cancerous and adjacent non-cancerous samples from three patients) were analysed.

## Data description

Table 1 summarises our study data. The specimens analysed in this here were taken from three patients who underwent surgery at Nippon Medical School Musashi Kosugi Hospital (Kawasaki, Japan). Data file 7 summarises the patients’ clinical characteristics. Study protocols were conducted in accordance with the 1975 Declaration of Helsinki and informed consent was obtained from each patient. The primary surgical specimens were evaluated following the World Health Organisation (WHO) 4th edition criteria. RNA preparation and microarray analysis in this study were performed as described previously [8]. Total RNA was extracted with a guanidinium thiocyanate/acid phenol–chloroform extraction method using RNAiso-Plus (Takara Bio, Kusatsu, Japan). The concentrations and A_260_/A_280_ ratios of RNA were determined using a NanoDrop 1000 spectrophotometer (Thermo Fisher Scientific) (Data file 8). The size distribution of total RNA was evaluated using Agilent TapeStation (Data file 9).

**Table 1:** Overview of data files/data sets

Label	Name of data file/data set	File types (file extension)	Data repository and identifier (DOI or accession number)
Data file 1	GSM5201868_Clariom_D_Human.CEL.gz	CEL file	Gene Expression Omnibus https://identifiers.org/geo:GSM5201868 [10]
Data file2	GSM5201869_Clariom_D_Human.CEL.gz	CEL file	Gene Expression Omnibus https://identifiers.org/geo:GSM5201869 [11]
Data file 3	GSM5201870_Clariom_D_Human.CEL.gz	CEL file	Gene Expression Omnibus https://identifiers.org/geo:GSM5201870 [12]
Data file 4	GSM5201871_Clariom_D_Human.CEL.gz	CEL file	Gene Expression Omnibus https://identifiers.org/geo:GSM5201871 [13]
Data file 5	GSM5201872_Clariom_D_Human.CEL.gz	CEL file	Gene Expression Omnibus https://identifiers.org/geo:GSM5201872 [14]
Data file 6	GSM5201873_Clariom_D_Human.CEL.gz	CEL file	Gene Expression Omnibus https://identifiers.org/geo:GSM5201873 [15]
Data file 7	Clinical features	Spreadsheet (.xlsx)	Mendeley Data (http://dx.doi.org/10.17632/9t595rjgsv.1) [16]
Data file 8	RNA quality	Spreadsheet (.xlsx)	Mendeley Data (http://dx.doi.org/10.17632/46x6vx5r4z.2) [17]
Data file 9	Size distribution of total RNA	Image (.pdf)	Mendeley Data (http://dx.doi.org/10.17632/3kpn7w49xv.2) [18]
Data file 10	Box plots of the signals from the microarray	Image (.pdf)	Mendeley Data (http://dx.doi.org/10.17632/rzg7tvkg2r.2) [19]
Data file 11	Scatterplots (carcinoma vs normal)	Image (.pdf)	Mendeley Data (http://dx.doi.org/10.17632/rd6k8b5xz4.1) [20]
Data file 12	Normalised signal values	Spreadsheet (.xlsx)	Mendeley Data (http://dx.doi.org/10.17632/w6p4bkhz25.1) [21]

The isolated RNA was subjected to microarray analysis using the human Affymetrix Clariom D platform (Thermo Fisher Scientific), a next-generation microarray device covering > 540,000 transcripts including long non-coding RNAs. The RNA samples were then labelled using the reagents and enzymes supplied in the GeneChip® WT Pico Reagent Kit (Thermo Fisher Scientific) according to the manufacturer’s instructions with slight modification. Briefly, total RNA (100 ng) from each sample was subjected to reverse-transcription and subsequent polymerase chain reaction to synthesise T7 promoter-tagged double-stranded cDNA. The cDNA was then subjected to in vitro transcription with T7 RNA polymerase to synthesize complementary RNA (cRNA). cRNA was reverse-transcribed using random primers to synthesize sense-strand cDNA.

After removing the template RNA using RNase H, sense-strand cDNA (5.5 μg) was digested with uracil-DNA glycosidase into fragments with sizes ranging from 40 to 70 nt. The success of fragmentation was confirmed using an Agilent 2100 Bioanalyzer. The fragments of cDNA were then labelled with biotin using terminal deoxynucleotidyl transferase and subjected to hybridisation according to the manual of GeneChip® WT Pico Reagent Kit. The Clariom D microarray was processed through the automatic washing step using a GeneChip® Hybridisation, Wash, and Stain Kit (Thermo Fisher Scientific) and GeneChip® Fluidics Station 450 (Thermo Fisher Scientific). Hybridised targets were stained with kit-provided streptavidin–phycoerythrin. Fluorescent signals from them were detected using a Scanner 3000 7G (Thermo Fisher Scientific). Raw data. i.e., CEL files, were produced using Affymetrix GeneChip Command Console Software and subjected to data processing using Affymetrix Expression Console Software. The CEL files were registered as datasets under Gene Expression Omnibus (GEO) accession no. GSE169393. A detection call algorithm was applied to filter and remove missing expression values based on absent/present calls. Using this algorithm, present, marginal, or absent calls were obtained for each probe set in each microarray. A scaling factor was applied to the normalised data from the CEL files to bring the average intensity for all probes on the microarray to 500, generating CHP files for use in Microarray Suite 5. For gene expression comparisons, data assigned to absent calls were omitted. The box plots of the microarray signals are available in Data file 10. The correlation of carcinoma and non-carcinoma signal values is available in Data file 11. Normalised signal values for individual genes are listed in Data file 12.

## Limitations

Here we describe DCIS transcriptomic profiling results, which may also provide insight regarding presurgical diagnostic biomarkers. One limitation of our study is that specimens were isolated by surgical resection. Therefore, the applicability of the results to specimens obtained by core-needle aspiration biopsy should be validated as described previously [9]. Another limitation is the small sample size. Furthermore, qRT-PCR analysis should be conducted to validate the differential gene expression patterns identified here.

## Data Availability

The raw microarray data described in this article can be freely and openly accessed on the Gene Expression Omnibus (GEO) database under the accession number GSE169393 [10–15]. The image and table data described in this article and the detailed methodology description are available from Mendeley Data [16–21]. Please see Table 1 for details and links to the data.

## Abbreviations

DCIS: Ductal carcinoma in situ
GEO: Gene expression omnibus
SPC: Solid papillary carcinoma

## References

[1] American Cancer Society. In: Breast Cancer Facts & Figures 2017–2018. American Cancer Society; 2017. https://www.cancer.org/content/dam/cancer-org/research/cancer-facts-and-statistics/breast-cancer-facts-and-figures/breast-cancer-facts-and-figures-2017-2018.pdf. Accessed 5 Aug 2021.

[2] Fentiman IS (1989). The treatment of in situ breast cancer. Acta Oncol.

[3] Narod SA, Iqbal J, Giannakeas V, Sopik V, Sun P (2015). Breast cancer mortality after a diagnosis of ductal carcinoma in situ. JAMA Oncol.

[4] Sagara Y, Mallory MA, Wong S, Aydogan F, DeSantis S, Barry WT (2015). Survival benefit of breast surgery for low-grade ductal carcinoma in situ: a population-based cohort study. JAMA Surg.

[5] Collins LC, Tamimi RM, Baer HJ, Connolly JL, Colditz GA, Schnitt SJ (2005). Outcome of patients with ductal carcinoma in situ untreated after diagnostic biopsy: results from the Nurses’ Health Study. Cancer.

[6] Sanders ME, Schuyler PA, Dupont WD, Page DL (2005). The natural history of low-grade ductal carcinoma in situ of the breast in women treated by biopsy only revealed over 30 years of long-term follow-up. Cancer.

[7] Brennan ME, Turner RM, Ciatto S, Marinovich ML, French JR, Macaskill P (2011). Ductal carcinoma in situ at core-needle biopsy:meta-analysis of underestimation and predictors of invasive breast cancer. Radiology.

[8] Jikuzono T, Ishikawa T, Hirokawa M, Sugitani I, Ishibashi O (2020). Microarray analysis of formalin-fixed, paraffin-embedded follicular thyroid carcinoma samples from patients who developed postoperative distant metastasis. BMC Res Notes.

[9] Jikuzono T, Horikawa A, Ishikawa T, Hirokawa M, Sugitani I, Inui T (2018). Proteinase K treatment improves RNA recovery from thyroid cells fixed with liquid-based cytology solution. BMC Res Notes.

[10] Gene Expression Omnibus; 2021. https://identifiers.org/geo:GSM5201868.

[11] Gene Expression Omnibus; 2021. https://identifiers.org/geo:GSM5201869.

[12] Gene Expression Omnibus; 2021. https://identifiers.org/geo:GSM5201870.

[13] Gene Expression Omnibus; 2021. https://identifiers.org/geo:GSM5201871.

[14] Gene Expression Omnibus; 2021. https://identifiers.org/geo:GSM5201872.

[15] Gene Expression Omnibus; 2021. https://identifiers.org/geo:GSM5201873.

[16] Ishibashi O (2021). Mendeley Data.

[17] Ishibashi O (2021). Mendeley Data.

[18] Ishibashi O (2021). Mendeley Data.

[19] Ishibashi O (2021). Mendeley Data.

[20] Ishibashi O (2021). Mendeley Data.

[21] Ishibashi O (2021). Mendeley Data.

